# An observational pragmatic quality-of-life study on paediatric tonsillectomy and waiting for surgery

**DOI:** 10.1007/s00405-022-07659-2

**Published:** 2022-09-22

**Authors:** Julie Huynh, Charmaine M. Woods, Eng H. Ooi

**Affiliations:** 1 Flinders Health and Medical Research Institute, College of Medicine and Public Health, Flinders University, Adelaide, Australia; 2 Department of Otolaryngology, Head and Neck Surgery, Flinders Medical Centre, Adelaide, Australia

**Keywords:** Tonsillectomy, Quality of life, Child, Sleep disordered breathing, Tonsillitis, Sleep apnoea

## Abstract

**Purpose:**

To investigate parental perceptions of the effects of tonsillectomy on their child’s quality of life while awaiting and following surgery in an Australian public health system.

**Methods:**

An observational pragmatic study was undertaken at a tertiary Australian hospital. Parents of paediatric patients (2–16 years of age) listed for tonsillectomy completed a validated quality-of-life questionnaire (T-14 Paediatric Throat Disorders Outcome Test) at the initial consultation, on day of surgery, 6 weeks post-operatively and 6 months post-operatively. T-14 scores were compared using the Related-Samples Wilcoxon Signed Rank Test.

**Results:**

Parents of 167 children participated in this study. There was a median wait time of 174 days (IQR 108–347) from the initial consultation until the day of surgery, with no significant change in median T-14 scores (35 [IQR 22–42] vs 36 [IQR 22–42]; *n* = 63; *p* > 0.05). There was a significant decrease from pre-operative T-14 scores to 6 weeks post-operatively (33.5 [IQR 22–42] vs 2 [IQR 0–5]; *n* = 160; *p* < 0.001), and this was sustained with a minor improvement at 6 months post-operatively (6 weeks 2 [IQR 0–5] vs 6 months 0 [IQR 0–2]; *n* = 148; *p* < 0.001).

**Conclusions:**

Paediatric tonsillectomy improves quality of life with a sustained benefit in the long term. There is no improvement to the patient’s quality of life while awaiting tonsillectomy, thus patient welfare can be improved through reducing waiting times for surgery.

**Supplementary Information:**

The online version contains supplementary material available at 10.1007/s00405-022-07659-2.

## Introduction

Paediatric tonsillectomy is a commonly performed surgical procedure. In Australia in 2017–2018, paediatric tonsillectomy accounted for 750 hospitalisations per 100,000 [1]. The main indications include sleep disordered breathing (SDB) and recurrent tonsillitis (RT) [2, 3]. These conditions adversely impact the child’s quality of life (QoL) and behaviour. Furthermore, there are consequential effects on the child’s parents and the health system, such as days off school translating to days off work for their parents, and an increase in presentations at primary care clinics and emergency rooms [4–6].

Australia has a mixed public and private healthcare system, where the former is entirely government-funded, whereas the latter is government-subsidised with the remainder paid by the patient and their private health insurer. In 2017–2018, the median waiting time for tonsillectomy (after initial specialist consultation) in an Australian public hospital was 121 days [7]. Private hospitals in Australia perform 50% more tonsillectomies than public hospitals, yet public patients wait almost 3 times longer [8, 9]. This highlights an inequality in healthcare access, where there are higher rates of tonsillectomies in patients of higher socioeconomic status despite higher rates of SDB and RT in patients of a lower socioeconomic status [10].

This study aimed to evaluate changes in QoL of paediatric patients while awaiting tonsillectomy in an Australian public hospital, following recovery from surgery, and long term.

## Methods

This manuscript has been prepared with reference to the STROBE checklist for observational studies [11].

Ethical approval was obtained from the Southern Adelaide Clinical Human Research Ethics Committee (Project number 123.17, HREC reference: HREC/17/SAC/206, approved 31 July 2017). Participants were recruited consecutively from August 2017 to August 2018 during preadmission clinic or on the day of surgery. Parents provided written, informed consent.

An observational study was undertaken in paediatric patients placed on the elective surgical waiting list for a tonsillectomy in a public tertiary hospital in South Australia. Inclusion criteria included: age 2–16 years; undergoing a tonsillectomy with or without an adenoidectomy and/or grommet insertion as an elective procedure for sleep disordered breathing and/or recurrent tonsillitis [12]. Exclusion criteria comprised of: non-English speaking parents (tool used only validated in English); undergoing an emergency procedure; undergoing tonsillectomy combined with additional procedure (excluding adenoidectomy/grommet insertion); cranio-facial anomalies—congenital or acquired; diagnosed with a neuromuscular disorder, a recognised congenital syndrome, a coagulation/bleeding disorder, and/or severe laryngomalacia; malignancy; tracheostomy in situ.

The T-14 Paediatric Throat Disorders Outcome Test was utilised to measure the patient’s symptoms and health-related QoL via parental reports [13]. This validated, sensitive tool was developed by Hopkins et al. in 2010 for use in the UK population and was adapted for use in the Australian population (Supplementary Material) [14]. It is comprised of fourteen Likert-scale questions, with a total score calculated (range 0–70), where a higher score signifies greater burden of symptoms and lower health-related QoL (mean score of healthy controls is 0.78 [range 0–11]) [13]. The T-14 also comprises of two sub-scores, with questions 1–6 totalling for an ‘obstruction’ symptom sub-score and questions 7–14 totalling for an ‘infection’ symptom sub-score.

Administration of the T-14 questionnaire to parents is part of the standard clinical process during the initial consultation clinic and responses were extracted from the patients’ medical chart. While awaiting surgery, all patients received ongoing medical management from their primary care physician. The T-14 questionnaire was also administered on day of surgery, at 6-week post-operative (the standard timeframe for post-operative review at this institution as surgical recovery is assessed as completed), and at 6 months. T-14 questionnaires were administered either in person or via telephone call from an ENT doctor or member of the research team.

Data collected included: age; gender (male, female); surgical indication (SDB, RT, both); date of initial consultation, surgery, post-operative review, and 6-month follow-up; Brodsky Tonsil Grade (1–4, a scale to describe tonsil size)[15]; surgical procedure (tonsillectomy, adenotonsillectomy, tonsillectomy and grommets, adenotonsillectomy and grommets); and T-14 scores (at initial consultation, at surgery, 6-week post-operatively, and 6 months post-operatively). T-14 total scores and sub-scores were calculated for each timepoint.

### Data analysis

T-14 data were not normally distributed and, therefore, presented as medians with interquartile ranges (IQR). A sub-group analysis of children (age 2–4 years) undergoing tonsillectomy for SDB was undertaken. Statistical analysis utilised non-parametric tests, specifically T-14 scores were compared using the Related-Samples Wilcoxon Signed Rank Test. Statistical analysis utilised IBM Corp. Released 2017. IBM SPSS Statistics for Windows, Version 25.0. Armonk, NY: IBM Corp. Graphs were generated in Prism 9.0™ (GraphPad Software, Inc, La Jolla, CA, USA).

## Results

### Patient demographics

All caregivers for 167 patients (94 males and 73 females, median age of 5.19 years) completed the pre-operative T-14 questionnaire at the time of surgery (Table 1). T-14 questionnaire data were available from the time of the initial consultation for 63 participants. Post-operative T-14 questionnaires were collected from 160 and 148 participants at 6 weeks and 6 months, respectively, with an overall drop-out rate of 12%. The demographics of the patients, surgical indications for tonsillectomy, Brodsky tonsil grade, surgical techniques and surgical waiting times are presented in Table 1. Patients in this study included those who are culturally and linguistically diverse and all were accompanied by at least one parent able to read or converse in English. Duration of waiting times for surgery was median 174 (IQR 7–529) days, with 85% of patients waiting longer than 90 days for surgery (median 184 [IQR 146.5–379] days). Three patients had their surgery expedited due to worsening symptoms (failure to thrive, repeated hospital admissions for treatment of recurrent tonsillitis, and worsening obstructive sleep apnoea).

**Table 1:** Patient demographics, waiting time for tonsillectomy, and surgical details

Demographics	Count, *n* (%)
*n*	167
Male:female	94:73 (56%:44%)
Age, years (median [range])	5 [2–16]
Indication	
Sleep Disordered Breathing	108 (65%)
Recurrent tonsillitis	8 (5%)
Both	51 (31%)
Waiting time for tonsillectomy	
Duration, days (median [IQR])	174 [IQR 7–529]
< 90-day duration, days (median [IQR])	*n* = 25 (15%) 69 [63–81]
≥ 90-day duration, days (median [IQR])	*n* = 142 (85%) 184 [147–379]
Brodsky Tonsil Grade	
1	3 (2%)
2	26 (16%)
3	106 (63%)
4	32 (19%)
Surgical procedure	
Tonsillectomy	2 (1%)
Adenotonsillectomy	121 (72%)
Adenotonsillectomy and grommets	43 (26%)
Tonsillectomy and grommets	1 (1%)
Surgical tonsillectomy technique	
BiZact	97 (58%)
Cold steel	56 (34%)
Bipolar diathermy	14 (8%)

### T-14 outcomes

**Fig. 1:**
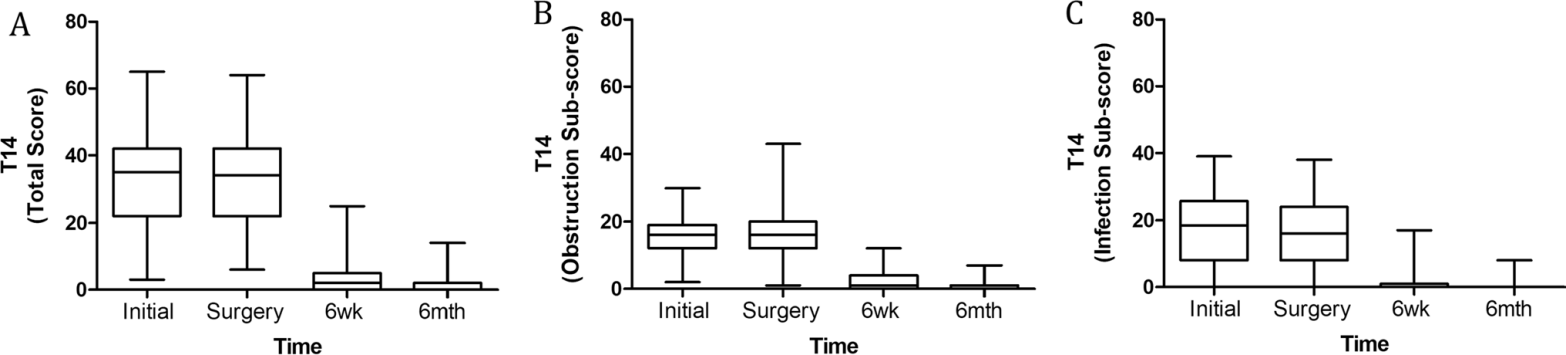
Parental perspective of child’s quality of life while waiting for and following tonsillectomy. T-14 total scores (**A**), obstructive sub-scores (**B**) and infection sub-scores (**C**) pre-operatively at initial consultation (*n* = 63), day of surgery (*n* = 167), at 6 weeks post-operatively, (*n* = 160), and 6 months post-operatively (*n* = 148)

Changes in QoL as measured using the T-14 are presented in Fig. 1. Over the waiting period between initial consultation and day of surgery, there was no statistically significant difference in median total T-14 scores (35 [IQR 22–42] vs 36 [IQR 22–42]; *n* = 63; *p* > 0.05). Following surgery there was a significant decrease in median total T-14 scores at the 6-week post-operative follow-up (33.5 [IQR 22–42] vs 2 [IQR 0–5]; *n* = 160; *p* < 0.001). At 6-month follow-up there was a further reduction in median total T-14 score (6-week median of 2 [IQR 0–5] vs 6-month median of 0 [IQR 0–2]; *n* = 148; *p* < 0.001), with T-14 scores at a normal range [13]. Similarly, median T-14 obstructive and infection sub-scores also demonstrated no significant change, while they were waiting for elective tonsillectomy (obstructive: initial 16 [IQR 12–19] vs day of surgery 16 [IQR 12–21]; *n* = 63; *p* > 0.05) (infection: initial 19 [IQR 8–26] vs day of surgery 17 [IQR 6–26]; *n* = 63; *p* > 0.05), but significant improvement at 6 weeks (obstructive: day of surgery 16 [IQR 12–20] vs 6 weeks 1 [IQR 0–4]; *n* = 160; *p* < 0.0001) (infection: day of surgery 16 [IQR 8–24] vs 6 weeks 0 [IQR 0–1]; *n* = 160; *p* < 0.0001), which was sustained with further minor improvement at 6 months post-operatively (obstructive: 6 weeks 1 [IQR 0–4] vs 6 months 0 [IQR 0–0]; *n* = 148; *p* < 0.0001) (infection: 6 weeks 0 [IQR 0–1] vs 6 months 0 [IQR 0–1]; *n* = 148; *p* < 0.0001).

**Fig. 2:**
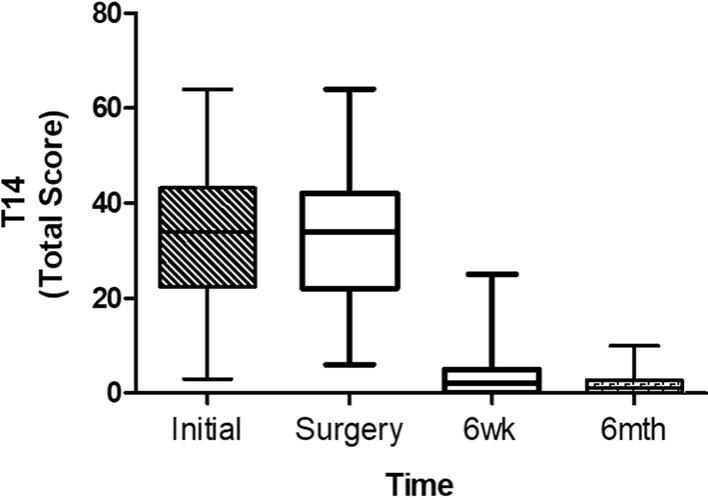
Parental perspective of young child’s quality of life with sleep disordered breathing while waiting for and following tonsillectomy. Children aged 2–4 years with a surgical indication including sleep disordered breathing (*n* = 28). T-14 total scores (**A**) and T-14 obstruction scores (**B**) pre-operatively at initial consultation, day of surgery, at 6 weeks post-operatively, and 6 months post-operatively

A sub-group analysis of children aged between 2 and 4 years, undergoing tonsillectomy for SDB was undertaken (*n* = 28; Fig. 2). This sub-group waited a median of 134 (IQR 94–205) days for surgery from initial consultation. The median T-14 scores at initial consultation compared to day of surgery showed no significant change (34 [IQR 22–43] vs 33 [IQR 22–40]; *n* = 63; *p* = 0.4224). The median T-14 scores significantly decreased from initial consultation to 6 months post-operatively (34 [IQR 22–43] vs 1 [IQR 0–3]; *n* = 28; *p* < 0.0001). A similar response was observed for T-14 obstructive sub-scores (initial 18 [IQR 14–21] vs day of surgery 18.5 [IQR 13.25–22]; *p* > 0.05) (day of surgery 18.5 [IQR 13.25–22] vs 6 months 1 [IQR 0–2]; *p* < 0.0001).

## Discussion

SDB and RT, both have significant impacts to the health and QoL of children, their families, and the healthcare system [4, 5]. Tonsillectomy is a surgical treatment with reported efficacy for improving the symptom burden of children with SDB and/or RT [2]. However, tonsillectomy is generally considered a non-urgent elective procedure resulting in a large discrepancy in waiting times for paediatric tonsillectomy between the patients of the Australian public and private healthcare systems [1]. This observational study demonstrates that a child’s QoL remains adversely impacted while waiting for elective tonsillectomy, which was often 6 months or longer, for the majority of patients undergoing treatment in a public hospital.

Children’s QoL, adversely impacted while waiting for paediatric tonsillectomy, demonstrated minimal improvement in T-14 scores in those that met the indications for surgery [2, 16]. However, surgery significantly improved QoL by 6 weeks post-operatively and this improved QoL was maintained at 6 months post-operatively [13]. Analysis of the T-14 infection and obstruction sub-scores showed similar results with no improvement while waiting for surgery. The group of children waiting for surgery could represent a ‘watchful waiting’ group, who ultimately had improved T-14 scores after surgery and importantly, the improvement was sustained at 6 months post-operatively. These findings highlight the health benefit of paediatric tonsillectomy to the child’s QoL and raises the importance of reducing wait times for elective surgery in the Australian public healthcare system.

These study outcomes are supported by other investigations using the T-14 questionnaire. The present study is supported by Hopkins et al. [13] demonstrating, in a UK population, little change to QoL while awaiting tonsillectomy and a large improvement in QoL post-operatively that is sustained in the longer term. These findings are reinforced by Konieczny et al. who used the T-14 questionnaire to demonstrate the continued benefits of tonsillectomy at follow-up after 5 years [17]. Similar findings showing substantial improvements to QoL post-operatively were reported in the Childhood Adenotonsillectomy Trial (CHAT) and other randomised clinical trials (RCTs) [18–21].

The Karolinska Adenotonsillectomy (KATE) study was an RCT comparing adenotonsillectomy with 6 months watchful waiting for management of non-severe obstructive sleep apnoea (OSA) in 53 children aged 2–4 years [16]. The KATE study used the Obstructive Sleep Apnea-18 (OSA-18) questionnaire as the tool to measure QoL [22]. A sub-group analysis consisting of children of similar age, SDB and wait duration showed no improvement in QoL using the T-14 scores while waiting for surgery and we infer our study’s findings strongly support the KATE study findings and recommendations for treatment with paediatric tonsillectomy in these patients.

The strengths of this study include its pragmatic observational design, highlighting the effectiveness of paediatric tonsillectomy on patient’s QoL in routine real-life clinical practice [23]. Each participant served as their own control and were followed longitudinally to determine changes in their QoL. We have previously reported that the T-14 questionnaire has high compliance with parents and is suitable for Australian patients undergoing tonsillectomy, as well as its use in assessing clinical efficacy of the BiZact tonsillectomy device [14, 24]. This study has demonstrated that parents perceive their child’s symptoms do not significantly change while waiting for elective surgery. However, parents perceive a significant improvement in their child’s symptoms following tonsillectomy and that this benefit is sustained even at 6 months post-operatively. We can also infer that QoL while waiting for elective surgery in children who meet criteria for tonsillectomy does not improve, and therefore, shortening waiting times for tonsillectomy in an Australian public hospital system would benefit a child’s QoL. There are limitations in this study. Patients with SDB and/or RT are typically referred by the general practitioner to either a public hospital clinic or a private clinic. The waiting time for the public pathway to be seen in clinic is predictably longer, for example, 9-month median waiting time for an appointment in an ENT clinic in our institution or 14 months at the state’s children’s hospital [25], whereas the estimated time for a patient to be seen in a private clinic is generally shorter. Furthermore, the wait time in this study for surgery from initial consultation was a median of 174 days, compared to the national Australian public hospital median of 121 days [7]. We speculate the possible reasons for this include our tonsillectomy waitlist consisting of many low priority cases with few high priority cases. The low priority cases, triaged as Category 3 in our institution, are planned for surgery within 365 days and approximately two-thirds are allocated in this non-urgent category, hence the increased median wait time reported in this study. Furthermore, our institution faces a high demand being one of two public hospitals performing paediatric tonsillectomy in South Australia and services a large metropolitan and rural area. Thus, our wait time is longer than the national average, further supporting the need to expedite tonsillectomy to improve child quality of life. The reasons for and number of parents requesting removal of their child from the elective waiting list for tonsillectomy could not be determined, though common anecdotal reasons include improvement of symptoms, relocation to another public hospital catchment area, or undergoing tonsillectomy at a private institution. This may be a confounding factor for interpretation of this data if this occurred in large numbers.

## Conclusions

This study demonstrated that children awaiting paediatric tonsillectomy have impaired quality of life, which improved to levels comparable to healthy children following tonsillectomy and this was sustained long term. This study highlights both the benefit of tonsillectomy to a child’s health and well-being, as well as the need to address the long waiting times for patients to have their elective surgery in an Australian public hospital.

## Supplementary Information

Below is the link to the electronic supplementary material.

Supplementary file1 (DOCX 38 KB)

## Data Availability

The data that support the findings of this study are available on request from the corresponding author. The data are not publicly available due to privacy or ethical restrictions.

## References

[1] Australian Commission on Safety and Quality in Health Care and Australian Institute of Health and Welfare (2021). The fourth Australian Atlas of healthcare variation.

[2] A joint Position paper of the Paediatrics & Child Health Division of The Royal Australasian College of Physicians and The Australian Society of Otolaryngology, Head and Neck Surgery, 2008 Sydney.

[3] Mitchell RB, Archer SM, Ishman SL, Rosenfeld RM, Coles S, Finestone SA (2019). Clinical practice guideline: tonsillectomy in children (update). Otolaryngol Head Neck Surg.

[4] Todd CA, Bareiss AK, McCoul ED, Rodriguez KH (2017). Adenotonsillectomy for obstructive sleep apnea and quality of life: systematic review and meta-analysis. Otolaryngol Head Neck Surg.

[5] Venekamp RP, Hearne BJ, Chandrasekharan D, Blackshaw H, Lim J, Schilder AGM (2015). Tonsillectomy or adenotonsillectomy versus non-surgical management for obstructive sleep-disordered breathing in children. Cochrane Database Syst Rev.

[6] Leupe P, Hox V, Debruyne F, Schrooten W, Claes NV, Lemkens N (2012). Tonsillectomy compared to acute tonsillitis in children: a comparison study of societal costs. B-ENT.

[7] Australian Institute of Health and Welfare (2018). Elective surgery waiting times 2017–18: Australian hospital statistics.

[8] Australian Institute of Health and Welfare 2019. Admitted patient care 2017–18: Australian hospital statistics. Health services series no. 90. Cat. no. HSE 225. AIHW, Canberra

[9] Australian Institute of Health and Welfare (2017) Private health insurance use in Australian hospitals, 2006–07 to 2015–16: Australian hospital statistics. AIHW, Canberra

[10] Tran AHL, Horne RSC, Liew D, Rimmer J, Nixon GM (2019). An epidemiological study of paediatric adenotonsillectomy in Victoria, Australia, 2010–2015: changing indications and lack of effect of hospital volume on inter-hospital transfers. Clin Otolaryngol.

[11] von Elm E, Altman DG, Egger M, Pocock SJ, Gøtzsche PC, Vandenbroucke JP (2008). The Strengthening the Reporting of Observational Studies in Epidemiology (STROBE) statement: guidelines for reporting observational studies. J Clin Epidemiol.

[12] Paradise JL, Bluestone CD, Bachman RZ, Colborn DK, Bernard BS, Taylor FH (1984). Efficacy of tonsillectomy for recurrent throat infection in severely affected children. Results of parallel randomized and nonrandomized clinical trials. N Engl J Med.

[13] Hopkins C, Fairley J, Yung M, Hore I, Balasubramaniam S, Haggard M (2010). The 14-item Paediatric Throat Disorders Outcome Test: a valid, sensitive, reliable, parent-reported outcome measure for paediatric throat disorders. J Laryngol Otol.

[14] Lam ME, Woods CM, Du C, Milton T, Kao SS-T, Huynh J (2019). Outcomes using the T-14 symptom score for tonsillectomy in an Australian paediatric population. Australian J Otolaryngol.

[15] Brodsky L (1989). Modern assessment of tonsils and adenoids. Pediatr Clin N Am.

[16] Fehrm J, Nerfeldt P, Browaldh N, Friberg D (2020). Effectiveness of adenotonsillectomy vs watchful waiting in young children with mild to moderate obstructive sleep apnea: a randomized clinical trial. JAMA Otolaryngol Head Neck Surg.

[17] Konieczny KM, Pitts-Tucker TN, Biggs TC, Pringle MB (2019). A five-year follow-up observational study of the T-14 paediatric throat disorders outcome measure in tonsillectomy and adenotonsillectomy. Ann R Coll Surg Engl.

[18] Marcus CL, Moore RH, Rosen CL, Giordani B, Garetz SL, Gerry TH (2013). A randomized trial of adenotonsillectomy for childhood sleep apnea. N Engl J Med.

[19] Garetz SL, Mitchell RB, Parker PD, Moore RH, Rosen CL, Giordani B (2015). Quality of life and obstructive sleep apnea symptoms after pediatric adenotonsillectomy. Pediatrics.

[20] Borgström A, Nerfeldt P, Friberg D (2017). Adenotonsillotomy versus adenotonsillectomy in pediatric obstructive sleep apnea: an RCT. Pediatrics.

[21] Fehrm J, Nerfeldt P, Sundman J, Friberg D (2018). Adenopharyngoplasty vs adenotonsillectomy in children with severe obstructive sleep apnea: a randomized clinical trial. JAMA Otolaryngol Head Neck Surg.

[22] Kao SS, Peters MDJ, Dharmawardana N, Stew B, Ooi EH (2017). Scoping review of pediatric tonsillectomy quality of life assessment instruments. Laryngoscope.

[23] Barnish MS, Turner S (2017). The value of pragmatic and observational studies in health care and public health. Pragmat Obs Res.

[24] Stepan L, Huang L, Huynh J, Xie P, Woods CM, Ooi EH (2021). Health related quality of life T-14 outcomes for pediatric bizact tonsillectomy. Medicina (Kaunas).

[25] SA Health (2021). SA health specialist outpatient clinics waiting time report census date as at 31 March 2021.

