# Supplementary material for: An observational pragmatic quality-of-life study on paediatric tonsillectomy and waiting for surgery
Source: Eur Arch Otorhinolaryngol. 2022 Sep 22;280(2):885–90. doi: 10.1007/s00405-022-07659-2 (PMC9849282; doi:10.1007/s00405-022-07659-2)
Supplement: Supplementary file 1 — Supplementary file1 (DOCX 38 KB) [file 405_2022_7659_MOESM1_ESM.docx]

# Supplementary Material

**Table 1. Pre-surgery T-14 Questionnaire (Flinders modification)**

| Considering how severe the problem is when your child experiences it & how often it has happened **over the last 6 months** for your child, please rate each item on how ‘bad’ it is by circling the number that corresponds with how you feel using this scale: | | | | | | |
| --- | --- | --- | --- | --- | --- | --- |
|  | **No problem** | **Very mild problem** | **Mild or slight problem** | **Moderate problem** | **Severe problem** | **Problem as bad as it can be** |
| 1. Snoring loudly during sleep | 0 | 1 | 2 | 3 | 4 | 5 |
| 1. Irregular or stopped breathing (apnoea) during sleep | 0 | 1 | 2 | 3 | 4 | 5 |
| 1. Daytime sleepiness | 0 | 1 | 2 | 3 | 4 | 5 |
| 1. Noisy breathing during the day | 0 | 1 | 2 | 3 | 4 | 5 |
| 1. Breathing through the mouth during the day | 0 | 1 | 2 | 3 | 4 | 5 |
| 1. Problems with poor appetite or poor eating habits (choking on food etc) | 0 | 1 | 2 | 3 | 4 | 5 |
| 1. Frequent earache or ear infections | 0 | 1 | 2 | 3 | 4 | 5 |
| 1. Repeated short term throat infections that last **less than 2 weeks** | 0 | 1 | 2 | 3 | 4 | 5 |
| 1. Constant or chronic throat infections that last **more than 2 weeks** | 0 | 1 | 2 | 3 | 4 | 5 |
| 1. Many phone calls to the GP | 0 | 1 | 2 | 3 | 4 | 5 |
| 1. Many visits to the family doctor or an Emergency department | 0 | 1 | 2 | 3 | 4 | 5 |
| 1. Taking antibiotics over & over for **less than 2 weeks** at a time | 0 | 1 | 2 | 3 | 4 | 5 |
| 1. Taking antibiotics for **more than 2 weeks** straight | 0 | 1 | 2 | 3 | 4 | 5 |
| 1. Missing school days due to sore throats | 0 | 1 | 2 | 3 | 4 | 5 |
| **TOTAL:** |  |  |  |  |  |  |
|  | | | | | | |
| Please enter *any further important symptoms t*hat occur as a result of your child’s throat problems that we have missed from the list above and give each a rating: | | | | | | |
|  | 0 | 1 | 2 | 3 | 4 | 5 |
|  | 0 | 1 | 2 | 3 | 4 | 5 |
|  | 0 | 1 | 2 | 3 | 4 | 5 |
| **TOTAL:** |  |  |  |  |  |  |
|  | | | | | | |
| **GRAND TOTAL** |  | | | | | |

**Table 2. Post-surgery T-14 Questionnaire (Flinders modification)**

| Considering how severe the problem is when your child experiences it & how often it has happened **over the last 4 weeks** for your child, please rate each item below on how ‘bad’ it is by circling the number that corresponds with how you feel using this scale: | | | | | | |
| --- | --- | --- | --- | --- | --- | --- |
|  | **No problem** | **Very mild problem** | **Mild or slight problem** | **Moderate problem** | **Severe problem** | **Problem as bad as it can be** |
| 1. Snoring loudly during sleep | 0 | 1 | 2 | 3 | 4 | 5 |
| 1. Irregular or stopped breathing (apnoea) during sleep | 0 | 1 | 2 | 3 | 4 | 5 |
| 1. Daytime sleepiness | 0 | 1 | 2 | 3 | 4 | 5 |
| 1. Noisy breathing during the day | 0 | 1 | 2 | 3 | 4 | 5 |
| 1. Breathing through the mouth during the day | 0 | 1 | 2 | 3 | 4 | 5 |
| 1. Problems with poor appetite or poor eating habits (choking on food etc) | 0 | 1 | 2 | 3 | 4 | 5 |
| 1. Frequent earache or ear infections | 0 | 1 | 2 | 3 | 4 | 5 |
| 1. Repeated short term throat infections that last **less than 2 weeks** | 0 | 1 | 2 | 3 | 4 | 5 |
| 1. Constant or chronic throat infections that last **more than 2 weeks** | 0 | 1 | 2 | 3 | 4 | 5 |
| 1. Many phone calls to the GP | 0 | 1 | 2 | 3 | 4 | 5 |
| 1. Many visits to the family doctor or an Emergency department | 0 | 1 | 2 | 3 | 4 | 5 |
| 1. Taking antibiotics over & over for **less than 2 weeks** at a time | 0 | 1 | 2 | 3 | 4 | 5 |
| 1. Taking antibiotics for **more than 2 weeks** straight | 0 | 1 | 2 | 3 | 4 | 5 |
| 1. Missing school days due to sore throats | 0 | 1 | 2 | 3 | 4 | 5 |
| **TOTAL:** |  |  |  |  |  |  |
|  | | | | | | |
| Please enter *any further important symptoms t*hat occur as a result of your child’s throat problems that we have missed from the list above and give each a rating: | | | | | | |
|  | 0 | 1 | 2 | 3 | 4 | 5 |
|  | 0 | 1 | 2 | 3 | 4 | 5 |
|  | 0 | 1 | 2 | 3 | 4 | 5 |
| **TOTAL:** |  |  |  |  |  |  |
|  | | | | | | |
| **GRAND TOTAL** |  | | | | | |
